# Ring neurons in the *Drosophila* central complex act as a rheostat for sensory modulation of aging

**DOI:** 10.1371/journal.pbio.3002149

**Published:** 2023-06-13

**Authors:** Christi M. Gendron, Tuhin S. Chakraborty, Cathryn Duran, Thomas Dono, Scott D. Pletcher

**Affiliations:** Department of Molecular and Integrative Physiology and Geriatrics Center, University of Michigan, Ann Arbor, Michigan, United States of America; University of Massachusetts Medical School, UNITED STATES

## Abstract

Sensory perception modulates aging, yet we know little about how. An understanding of the neuronal mechanisms through which animals orchestrate biological responses to relevant sensory inputs would provide insight into the control systems that may be important for modulating lifespan. Here, we provide new awareness into how the perception of dead conspecifics, or death perception, which elicits behavioral and physiological effects in many different species, affects lifespan in the fruit fly, *Drosophila melanogaster*. Previous work demonstrated that cohousing *Drosophila* with dead conspecifics decreases fat stores, reduces starvation resistance, and accelerates aging in a manner that requires both sight and the serotonin receptor 5-HT2A. In this manuscript, we demonstrate that a discrete, 5-HT2A-expressing neural population in the ellipsoid body (EB) of the *Drosophila* central complex, identified as R2/R4 neurons, acts as a rheostat and plays an important role in transducing sensory information about the presence of dead individuals to modulate lifespan. Expression of the insulin-responsive transcription factor *foxo* in R2/R4 neurons and insulin-like peptides *dilp3* and *dilp5*, but not *dilp2*, are required, with the latter likely altered in median neurosecretory cells (MNCs) after R2/R4 neuronal activation. These data generate new insights into the neural underpinnings of how perceptive events may impact aging and physiology across taxa.

## Abstract 2

Co-housing Drosophila with dead conspecifics accelerates aging, but how? This study reveals the neural mechanisms that modulate organismal aging in response to a sensory event, involving 5-HT2A-expressing neurons in the ellipsoid body of the Drosophila central complex.

## Introduction

A range of sensory processes, from the relatively simple (e.g., water, taste, or smell of food) to the more complex (e.g., the detection of the opposite sex), influences how long an organism lives as well as its health and vitality throughout life. Most of the research in this area has used simple model systems, such as *Drosophila melanogaster* or *Caenorhabditis elegans*, and has focused on identifying environmental cues with potent effects on aging or the sensory receptors and neurons that are involved in their initial detection [1]. Because peripheral sensory systems have been tuned by species-specific ecological conditions and evolutionary histories, these achievements are unlikely to provide direct insight into similar process in other systems, including humans. Nevertheless, they provide the necessary foundation for considering the more difficult task of understanding which neurons and associated neural states are influenced by sensory perception, how they relate to one another, and how they mechanistically relate to aging and physiology.

With this in mind, we chose to investigate the neural circuits and central signaling processes that underlie the physiological effects associated with the perception of dead conspecifics, or death perception, in *Drosophila*. It has been shown that when live flies see, and to a lesser extent smell, an excess of dead flies in their environment, they become aversive to other flies, and they exhibit significant acute and chronic physiological changes, including rapid decreases in stored fat and starvation resistance as well as chronically increased mortality [2]. These effects are reminiscent of the behavioral and physiologic changes seen in other species exposed to similar circumstances, such as necrophoresis in eusocial insects, vocalization and corpse inspection in elephants, or increased glucocorticoid levels detected in nonhuman primates [3], suggesting similarity in the effector processes that are recruited in response to this perceptive event. Indeed, in *Drosophila*, the effects of death perception reportedly involve the highly conserved biogenic amine, serotonin, as well as neural signaling through one of its receptors, 5-HT2A. It remained unknown which 5-HT2A-expressing neurons, or the molecules expressed by these cells, are required to modulate the global physiologic and associated lifespan changes that are caused by death perception. Understanding the neural circuits through which death perception impacts these phenotypes may inform future work directed toward understanding the consequences associated with this, and perhaps other sensory experiences in individuals, including humans, and may provide insight into how specific neural states impact behavior and physiology.

Herein, we identify neural substrates and circuits that transduce sensory information from the perception of dead individuals to meaningful changes in lifespan. We identified a small subset of 5-HT2A-expressing neurons in the ellipsoid body (EB) of *Drosophila* (specifically R2/R4 neurons), a center of sensory information integration and motor coordination, that were both required and sufficient for the lifespan effects caused by death exposure. The transcription factor associated with insulin-signaling, *foxo*, was required in these neurons. We also discovered that the *Drosophila* insulin-like peptides (*dilp*) *3* and *5*, but not *dilp2*, were required to mediate lifespan effects due to death perception. *Dilp* regulation appeared after changes in R2/R4 neuron activity, however, suggesting that these peptides do not directly impact Foxo activity in these neurons. By contributing to an understanding of the physiological effects of death exposure and the biological mechanism(s) that drive them, our results may provide insight for treating individuals who are routinely exposed to stressful situations surrounding death, including active combat soldiers and first responders.

## Results

### Ellipsoid body neurons respond to the presence of dead conspecifics

**Fig 1:**
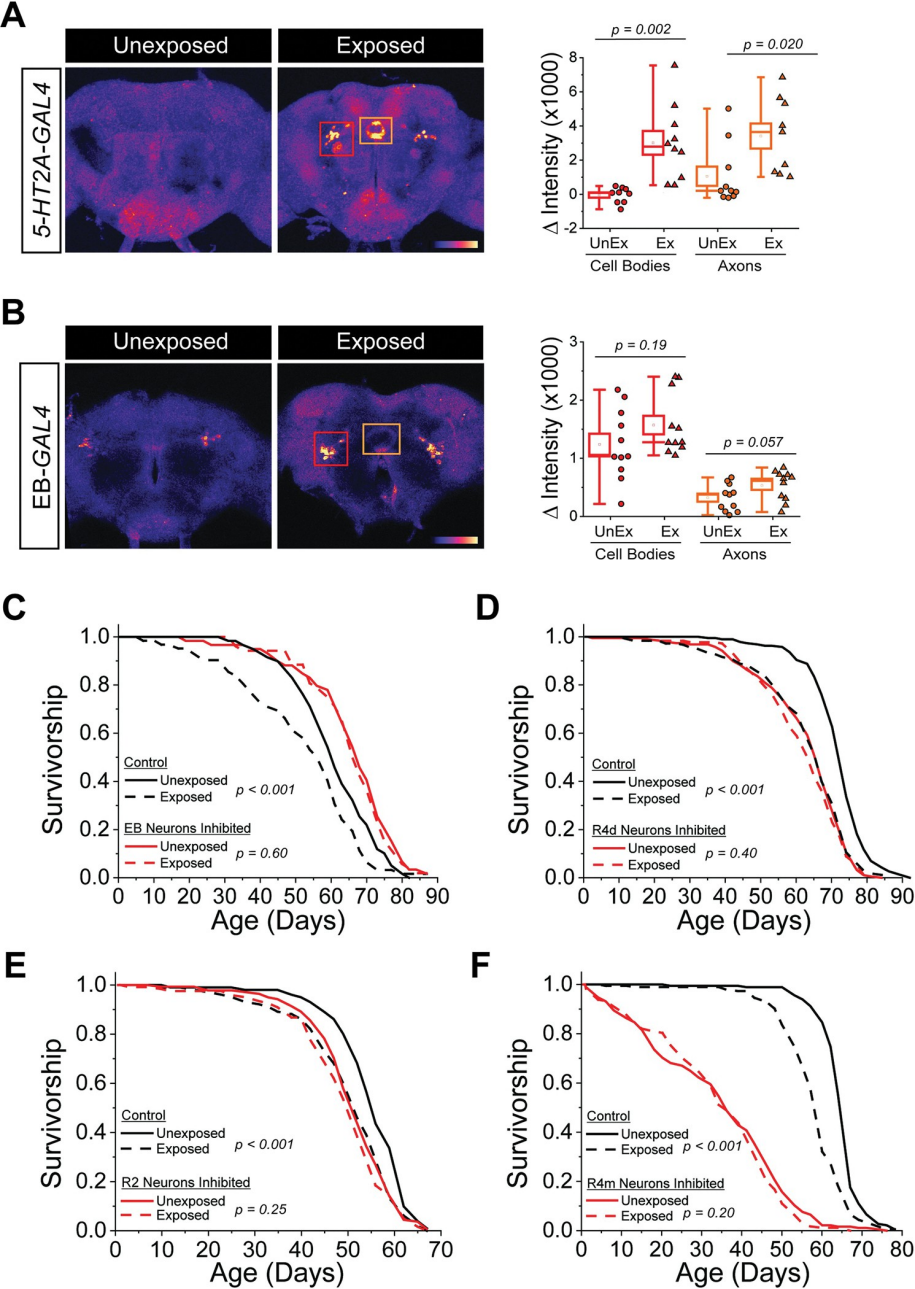
*5-HT2A*-expressing EB neurons show increased Ca^2+^ influx upon exposure to dead conspecifics and are required for lifespan changes due to death exposure. (A) The panel on the left are representative brain images from selective slices of *5-HT2A-GAL4* x NFAT unexposed and dead-exposed flies. The panel on the right is the quantification of the GFP-intensity seen in the ellipsoid cell bodies and axons from the regions indicated the picture of unexposed and dead-exposed brains. The pixel sum of all the stacks from the indicated region with background subtracted is shown. Each dot represents an individual brain (*n* = 10 brains for each treatment). (B) The panel on the left are representative brain images from selected slices of EB-*GAL4* x NFAT unexposed and dead-exposed flies. The panel on the right is the quantification of the GFP-intensity seen in the ellipsoid cell bodies and axons from the brain regions indicated in the picture of unexposed and dead-exposed brains. The pixel sum of all the stacks from the indicated regions with background subtracted is shown. Each dot represents an individual brain (*n* = 11 brains for each treatment). (C) Inhibiting EB neurons using EB-*GAL4* x UAS-*Kir*^*2*.*1*^ flies inhibited lifespan differences caused by exposure to dead individuals (*n* = 59 or 52 for unexposed or dead-exposed, respectively). Control flies used were EB-*GAL4* x *w*^*1118*^ (*n* = 59 or 62 for unexposed or dead-exposed, respectively). Inhibiting R4d (D), R2 (E), or R4m (F) neurons specifically also inhibited lifespan differences caused by exposure to dead individuals. The following genotypes were used: R4d-*GAL4* x UAS-*Kir*^*2*.*1*^(*n* = 189 for unexposed and *n* = 177 for dead-exposed), R4d-*GAL4* x *w*^*1118*^ (control; *n* = 187 for unexposed and *n* = 179 for dead-exposed), R2-*GAL4* x UAS-*GtACR1* (*n* = 139 for unexposed and *n* = 122 for dead-exposed), R2-*GAL4* x *w*^*1118*^ (control; *n* = 101 for unexposed and *n* = 103 for dead-exposed), R4m-*GAL4* x UAS-*GtACR1* (*n* = 190 for unexposed and *n* = 179 for dead-exposed), and R4m-*GAL4* x *w*^*1118*^ (control; *n* = 182 for unexposed and *n* = 193 for dead-exposed). Data for this figure can be found in the accompanying Supporting information (S1 Datasheet). EB, ellipsoid body; GFP, green fluorescence protein.

Having previously demonstrated that loss of the 5-HT2A receptor eliminated the ability of dead conspecifics to modulate lifespan [2], we sought to understand the underlying mechanisms involved. We focused our attention on the central nervous system (CNS) because we previously established that sight, and possibly olfaction, were involved in the response [2]. To identify individual neurons or neuronal populations that mediate the perceptive event or its consequences, we began by examining neural cell activation in 5-HT2A^+^ neurons using 5-HT2A-*GAL4* [4] and an NFAT-based tracing method (CaLexA) through which sustained neural activity drives expression of green fluorescence protein (GFP) [5]. We found that a short, 2-day exposure to freshly dead flies, which is known to affect the behavior of unexposed flies compared to dead-exposed individuals [2], led to a significant increase in fluorescent intensity relative to control animals in a population of neurons that was strongly indicative of the EB, suggesting that exposure to dead increased the activity of these neurons (Fig 1A). We observed a similar pattern when we expressed this same NFAT construct in EB neurons using the pan-EB-*GAL4* driver, R73A06 (hereafter EB*-GAL4*), although the result was marginally less significant than that observed using the 5-HT2A driver, likely because of higher background signal. Nevertheless, these data provide evidence that the activity of *5-HT2A*-expressing EB neurons are increased when flies are exposed to dead conspecifics (Fig 1B).

### R2 and R4 EB neuronal activity is required to alter lifespan when flies are co-housed with dead

We next tested whether EB neuronal activity was required to mediate the effects of death perception on lifespan. To accomplish this, we silenced these neurons by expressing an inward rectifier K+ channel (EB-*GAL4* > UAS-*Kir*^*2*.*1*^, [6]) and examined how this would impact the lifespan differences we saw when animals were exposed to dead compared to their unexposed controls. We observed that when EB neurons were silenced, the survivorship of flies was unaffected by the presence of dead (Fig 1C). Notably, silencing tubercular-bulbar (TuBu) neurons (GMR88A06-*GAL4* > UAS-*Kir*^*2*.*1*^), which form synaptic connections with EB neurons linked to visual feature detection [7], also abrogated the effect of death perception on survivorship (S1A and S1B Fig), an observation that is consistent with the reported involvement of sight in this phenotype [2].

The EB comprises a series of ring neurons, grouped into 11 subclasses based upon the staining of a global marker (DN-Cadherin) and *GAL4* driver lineage analysis [8]. We obtained *GAL4* driver lines that targeted 9 of these subclasses (R1, R2, R3a, R3d, R3p, R4m, R4d, R5, and R6; see S1 Table for the fly lines used) and tested whether silencing each group individually influenced the effect of death exposure on lifespan. We found that the survivorship of flies expressing *Kir*^*2*.*1*^ in R3a, R3p, R3d, R5, or R6 neurons remained significantly affected by the presence of dead conspecifics, indicating that activation of these neurons was not required for the effects of dead exposure on lifespan (Table 1). On the other hand, *Kir*^*2*.*1*^-mediated silencing of R4d neurons eliminated the significant effect on lifespan (Fig 1D). Expression of *Kir*^*2*.*1*^ using *GAL4* lines that targeted R1, R2, or R4m neurons resulted in developmental lethality, leading us to substitute optogenetic manipulations to induce their silencing after eclosion [9]. We observed that GtACR1-mediated, optogenetic silencing of R1 neurons did not influence the effect of death exposure on lifespan but that silencing of R2 and R4m led to partial and near-complete abrogation of lifespan effects, respectively (Fig 1E and 1F, and Table 1). Of note, we also tested several neuronal populations outside the EB for potential roles in this phenotype but observed no effect of inhibiting these neurons on the lifespan effects caused by the presence of dead (Table 2). We therefore conclude that the activity of R2 and R4 neurons are a specific and required component of the neural circuitry that modulates lifespan when flies are exposed to dead conspecifics.

**Table 1:** The R2, R4m, and R4d neuronal subsets of the EB are required to mediate the effects of cohousing with dead on lifespan.

		Dead-exposed treatment					Unexposed treatment						
Ring neurons	*N*	Mean (hrs)	Mean (days)	Median (hrs)	Median (days)	N2	Mean (hrs)	Mean (days)	Median (hrs)	Median (days)	Difference in mean	Difference in median	*p*-Value
R1*-GAL4* > UAS-*GtACR*	145	1,465.75	61.07	1,584.02	66.00	134	1,604.47	66.85	1,654.67	68.94	5.78	2.94	*p* = 0.049
R1*-GAL4* x *w*^*1118*^	134	1,583.19	65.97	1,654.67	68.94	125	1,711.15	71.30	1,751.02	72.96	5.33	4.01	*p* < 0.001
R2*-GAL4* > UAS*-GtACR*	122	1,181.12	49.21	1,246.09	51.92	139	1,227.13	51.13	1,246.09	51.92	1.92	0.00	*p* = 0.25
R2*-GAL4* x *w*^*1118*^	103	1,208.87	50.37	1,246.09	51.92	101	1,326.92	55.29	1,342.34	55.93	4.92	4.01	*p* < 0.001
R2*-GAL4* > UAS*-GtACR*	185	1,486.81	61.95	1,508.88	62.87	196	1,618.32	67.43	1,628.82	67.87	5.48	5.00	*p* < 0.001
R2*-GAL4* x *w*^*1118*^	203	1,418.46	59.10	1,413.99	58.92	187	1,608.93	67.04	1,628.82	67.87	7.94	8.95	*p* < 0.001
R3a*-GAL4* > UAS*-Kir*^2.1^	154	1,387.49	57.81	1,506.03	62.75	146	1,599.35	66.64	1,673.73	69.74	8.83	6.99	*p* < 0.001
R3a*-GAL4* x *w*^*1118*^	145	1,422.94	59.29	1,506.03	62.75	144	1,603.73	66.82	1,673.73	69.74	7.53	6.99	*p* < 0.001
R3p*-GAL4* > UAS*-Kir*^*2*.*1*^	154	766.99	31.96	715.33	29.81	152	1,091.62	45.48	1,170.14	48.76	13.53	18.95	*p* < 0.001
R3p*-GAL4* x *w*^*1118*^	140	1,161.39	48.39	1,218.64	50.78	154	1,561.33	65.06	1,601.92	66.75	16.66	15.97	*p* < 0.001
R3d*-GAL4* > UAS*-Kir*^*2*.*1*^	189	1,453.73	60.57	1,466.71	61.11	192	1,596.03	66.50	1,636.24	68.18	5.93	7.06	*p* < 0.001
R3d*-GAL4* x *w*^*1118*^	133	1,722.88	71.79	1,803.49	75.15	129	1,862.53	77.61	1,971.59	82.15	5.82	7.00	*p* < 0.001
UAS-*Kir2*.*1* x *w*^*1118*^	115	1,054.14	43.92	1,059.14	44.13	114	1,268.26	52.84	1,299.22	54.13	8.92	10.00	*p* < 0.001
R4d*-GAL4* > UAS*-Kir*^*2*.*1*^	177	1,486.18	61.92	1,561.99	65.08	189	1,506.22	62.76	1,611.66	67.15	0.83	2.07	*p* = 0.42
R4d*-GAL4* x *w*^*1118*^	179	1,509.49	62.90	1,611.66	67.15	187	1,731.97	72.17	1,729.65	72.07	9.27	4.92	*p* < 0.001
R4d*-GAL4* > UAS*-Kir*^*2*.*1*^	162	1,603.04	66.79	1,632.44	68.02	187	1,613.12	67.21	1,632.44	68.02	0.42	0.00	*p* = 0.26
R4d*-GAL4* x *w*^*1118*^	188	1,711.60	71.32	1,752.68	73.03	170	1,852.54	77.19	1,848.34	77.01	5.87	3.99	*p* < 0.001
R4d*-GAL4* > UAS*-Tetx*	163	1,404.70	58.53	1,440.03	60.00	178	1,424.22	59.34	1,440.04	60.00	0.81	0.00	*p* = 0.64
UAS-*TeTx* x *w*^*1118*^	176	1,210.71	50.45	1,223.99	51.00	165	1,339.81	55.83	1,392.53	58.02	5.38	7.02	*p* < 0.001
R4m*-GAL4* > UAS*-GtACR*	56	1,087.24	45.30	1,174.73	48.95	54	1,107.96	46.17	1,221.03	50.88	0.86	1.93	*p* = 0.20
R4m*-GAL4* x *w*^*1118*^	53	1,334.27	55.59	1,389.94	57.91	52	1,519.18	63.30	1,510.81	62.95	7.70	5.04	*p* < 0.001
R4m*-GAL4* > UAS*-GtACR*	179	821.58	34.23	868.89	36.20	190	828.74	34.53	868.89	36.20	0.30	0.00	*p* = 0.20
R4m*-GAL4* x *w*^*1118*^	193	1,399.26	58.30	1,441.50	60.06	182	1,561.81	65.08	1,608.98	67.04	6.77	6.98	*p* < 0.001
R5*-GAL4* x UAS-*Kir*^*2*.*1*^	116	978.47	40.77	1,011.59	42.15	111	1,225.71	51.07	1,179.54	49.15	10.30	7.00	*p* < 0.001
R5*-GAL4* x *w*^*1118*^	192	1,728.16	72.01	1,900.05	79.17	191	2,009.91	83.75	2,066.70	86.11	11.74	6.94	*p* < 0.001
R6*-GAL4* > UAS- *Kir*^*2*.*1*^	155	1,068.70	44.53	1,097.84	45.74	134	1,255.95	52.33	1,265.07	52.71	7.80	6.97	*p* = 0.011
R6*-Gal4* x *w*^*1118*^	142	1,282.69	53.45	1,338.49	55.77	132	1,404.68	58.53	1,386.88	57.79	5.08	2.02	*p* = 0.039

EB, ellipsoid body.

**Table 2:** Lifespan changes caused by death perception in specific fly mutants and/or flies where neural cell populations were affected.

Genotypes	Unexposed treatment					Exposed treatment							
	*N*	Mean (hrs)	Mean (days)	Median (hrs)	Median (days)	N	Mean (hrs)	Mean (days)	Median (hrs)	Median (days)	Difference (mean days)	Difference (median days)	*P*-Values
*CCKLR-17D1* ^*MB02688*^													
Control	199	1,118.3	46.6	N/A	N/A	195	986.7	41.1	N/A	N/A	5.5	N/A	*p* < 0.001
Mutant	196	1,120.1	46.7	N/A	N/A	180	1,032.5	43.0	N/A	N/A	3.6	N/A	*p* < 0.001
*Crz* Cell Activation													
Crz*-GAL4* x *w*^*1118*^	200	1,144.2	47.7	1,273.2	53.1	200	994.5	41.4	1,057.3	44.1	6.2	9.0	*p* < 0.001
Crz*-GAL4* > UAS-*NaChBac*	200	1,150.9	48.0	N/A	N/A	200	1,040.9	43.4	1,105.1	46.0	4.6	N/A	*p* < 0.001
Crz Cell Inactivation													
Crz*-GAL4* x *w*^*1118*^	197	1,095.3	45.6	1,130.4	47.1	200	929.5	38.7	889.1	37.0	6.9	10.1	*p* < 0.001
*w*^*1118*^ X UAS-*reaper*	194	974.5	40.6	962.1	40.1	198	859.7	35.8	889.1	37.0	4.8	3.0	*p* < 0.001
Crz*-GAL4* > UAS-*reaper*	181	1,027.1	42.8	1,057.3	44.1	185	877.3	36.6	962.1	40.1	6.2	4.0	*p* < 0.001
*Dh44 Full*													
Control	200	1,465.6	61.1	1,512.8	63.0	203	961.8	40.1	1,011.1	42.1	21.0	20.9	*p* < 0.001
Mutant	140	1,356.0	56.5	1,346.3	56.1	142	830.9	34.6	890.5	37.1	21.9	19.0	*p* < 0.001
*Dh44 Partial*													
Control	200	1,298.3	54.1	1,296.1	54.0	207	895.2	37.3	912.1	38.0	16.8	16.0	*p* < 0.001
Mutant	203	1,349.8	56.2	1,344.6	56.0	204	857.6	35.7	912.1	38.0	20.5	18.0	*p* < 0.001
*Dh44-R1*													
Control	200	1,169.8	48.7	N/A	N/A	200	1,039.2	43.3	N/A	N/A	5.4	N/A	*p* < 0.001
Mutant	200	1,205.1	50.2	N/A	N/A	200	1,114.1	46.4	N/A	N/A	3.8	N/A	*p* < 0.001
*Dop1R1* ^*f022676*^													
Control	172	1,113.8	46.4	1,105.8	46.1	124	806.0	33.6	768.5	32.0	12.8	14.1	*p* < 0.001
Mutant	194	1,086.8	45.3	1,105.8	46.1	176	739.2	30.8	768.5	32.0	14.5	14.1	*p* < 0.001
*dumb*													
Control	178	1,527.3	63.6	1,679.8	70.0	190	1,251.2	52.1	1,411.6	58.8	11.5	11.2	*p* < 0.001
Mutant	178	1,527.3	63.6	1,679.8	70.0	208	1,338.0	55.8	1,411.6	58.8	7.9	11.2	*p* < 0.001
*dunce*													
Control	121	1,578.8	65.8	1,629.9	67.9	121	801.7	33.4	787.5	32.8	32.4	35.1	*p* < 0.001
Mutant	121	1,346.9	56.1	1,340.6	55.9	127	1,125.2	46.9	1,172.2	48.8	9.2	7.0	*p* < 0.001
*Henna*													
Control	190	1,282.0	53.4	1,276.8	53.2	176	1,030.5	42.9	1,009.9	42.1	10.5	11.1	*p* < 0.001
Mutant	195	1,126.1	46.9	1,106.7	46.1	177	892.3	37.2	888.1	37.0	9.7	9.1	*p* < 0.001
*NPF* ^*Sk2*^													
Control	88	984.4	41.0	1,007.1	42.0	75	860.0	35.8	888.1	37.0	5.2	5.0	*p* < 0.001
Mutant	101	1,188.9	49.5	N/A	N/A	80	1,159.7	48.3	N/A	N/A	1.2	N/A	*p* < 0.001
*p38b*													
nSyb*-GAL4 x w*^*1118*^	102	1,438.3	59.9	1,560.7	65.0	112	1,327.1	55.3	1,344.5	56.0	4.6	9.0	*p* < 0.001
*w*^*1118*^ x UAS-*p38b*	112	1,519.1	63.3	1,609.3	67.1	107	1,295.7	54.0	1,275.0	53.1	9.3	13.9	*p* < 0.001
nSyb*-GAL4* > UAS-*p38b*	119	1,505.5	62.7	1,560.7	65.0	114	1,422.8	59.3	1,512.5	63.0	3.4	2.0	*p* < 0.001
*rutabaga*													
Control	178	1,527.3	63.6	1,679.8	70.0	190	1,251.2	52.1	1,411.6	58.8	11.5	11.2	*p* < 0.001
Mutant	199	1,612.2	67.2	1,679.8	70.0	199	1,338.0	55.8	1,411.6	58.8	11.4	11.2	*p* < 0.001
*Trh* Cell Inhibition													
Trh*-GAL4* x *w*^*1118*^	196	1,409.0	58.7	1,393.9	58.1	194	1,232.4	51.3	1,224.9	51.0	7.4	7.0	*p* < 0.001
Trh*-Gal4* > UAS-*TNT*	200	1,331.5	55.5	1,345.9	56.1	189	1,249.2	52.1	1,295.9	54.0	3.4	2.1	*p* < 0.001

### R2/R4 neuron activity is increased when flies are exposed to dead

**Fig 2:**
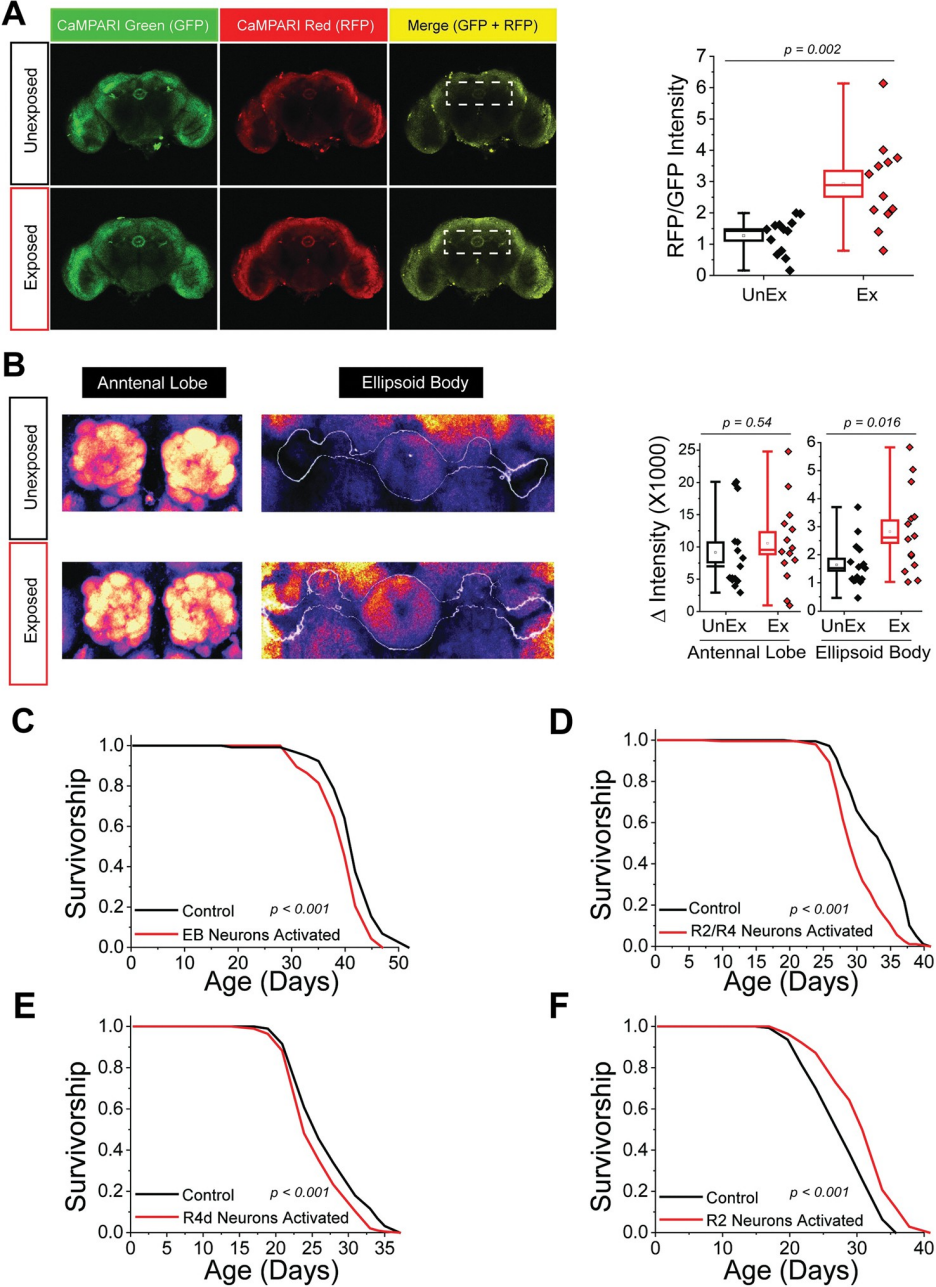
R2/R4 neurons show increased Ca^2+^ influx upon death exposure and were sufficient to alter fly lifespan when activated in the absence of dead. (A) Representative images of brains that express UAS-CaMPARI in R2/R4 neurons and their quantification from flies that were unexposed (*n* = 14) or exposed (*n* = 12) to dead conspecifics. (B) Representative false color images and quantification of the antennal lobe or the EB that were stained using an anti-Bruchpilot antibody from flies that were unexposed (*n* = 15) or exposed (*n* = 14) to dead conspecifics. Activation of EB neurons (C), R2/R4d (D), or R4d alone (E) in the absence of dead decreased fly lifespan, whereas activation of R2 (F) was sufficient to increase lifespan. The following genotypes were used: EB-*GAL4* x UAS-*TRPA1* (*n* = 163), EB-*GAL4* x *w*^*1118*^ (control; *n* = 117), R2/R4-*GAL4* x UAS-*TRPA1* (*n* = 196), R2/R4-*GAL4* x *w*^*1118*^ (control; *n* = 179), R4d-*GAL4* x UAS-*TRPA1* (*n* = 197), R4d-*GAL4* x *w*^*1118*^ (control; *n* = 190), R2-*GAL4* x UAS-*TRPA1* (*n* = 140), and R2-*GAL4* x *w*^*1118*^ (control; *n* = 140). Data for this figure can be found in the accompanying Supporting information (S2 Datasheet). EB, ellipsoid body.

If R2 and R4 neuronal activation is required for death perception to modulate lifespan, we might expect that the activity of these neurons would be increased when flies are exposed to dead. To determine whether this was indeed the case, we used the calcium-modulated photoactivatable ratiometric integrator system, CaMPARI, which results in Ca^2+^-dependent conversion of GFP to red fluorescence protein (RFP) in activated neurons when exposed to 405 nm light [10]. For this experiment, we used CaMPARI rather than the CaLexA system described above because of its ability to temporally constrain calcium measures and control for tonic activity, both of which limit unrelated signals at the potential cost of reduced signal. When we targeted expression of the CaMPARI protein to both R2 and R4 neurons (using SS02769-*GAL4*, hereafter R2/R4-*GAL4*; [11]) and induced conversion during exposure, we observed a significant increase in the relative RFP signal in flies exposed to dead compared to unexposed controls, indicating that their activity was significantly increased (Fig 2A). CaLexA experiments using flies that were kept in the dark during the exposure period revealed no significant intensity differences between dead-exposed and control flies, consistent with the notion that sight is required for this treatment to induce changes in neural activity (S1C Fig).

Alterations in the abundance of the synaptic active zone protein Bruchpilot (Brp) are thought to reflect underlying activity-dependent changes in neural synaptic structure that are generally associated with changes in synaptic strength [12]. We therefore examined the abundance of Brp in the brains of unexposed and dead-exposed flies and observed increased Brp antibody staining in the EB neurons of dead-exposed flies compared to unexposed animals but not in other brain regions such as the antennal lobe (Fig 2B). These data are consistent with changes in the activity of R2/R4 neurons and suggest that exposure to dead may also influence neural plasticity.

### Activation of R2/R4 neurons is sufficient to alter lifespan in the absence of dead conspecifics

If EB neurons are generally, or R2/R4 neurons more specifically, involved in mediating the effects of death perception on lifespan, we might expect that activating them in the absence of dead would be sufficient to affect lifespan. Expression of the transient receptor potential cation channel TRPA1 using EB-*GAL4* resulted in a significant lifespan decrease when flies were aged at 29°C, a temperature sufficient for channel activation, but not when flies were kept below the threshold temperature of 18°C (Figs 2C and S2). Expression of TRPA1 in R2/R4 neurons or in R4d neurons alone also reduced the lifespan of adults aged at the activating temperature (29°C; Fig 2D and 2E). To our surprise, TRPA1-mediated activation of R2 neurons alone significantly increased lifespan (Fig 2F). These data are in line with recent findings demonstrating that R2 and R4 neurons can differentially impact fly physiology and behavior, depending on the experimental context [13]. Altogether, we conclude that the EB, specifically R2/R4 neurons, are a dynamic component of the neural network that plays a prescriptive role in modulating aging following exposure to dead conspecifics.

### The 5-HT2A receptor is required on R2/R4 neurons to mediate the effects of death perception on lifespan

**Fig 3:**
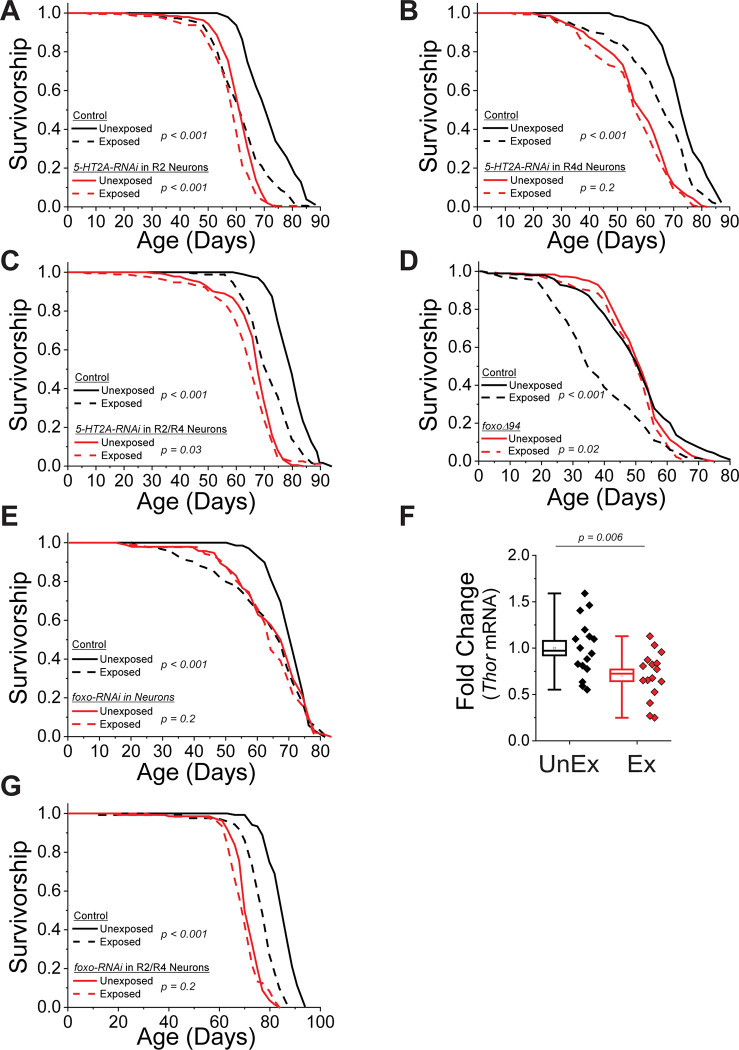
5-HT2A and Foxo are required in R2/R4 neurons to mediate the effects of death perception on lifespan. Knock-down of *5-HT2A* mRNA expression in R2 alone (A), in R4d alone (B), or in R2/R4 inhibited lifespan differences due to death perception. The following genotypes were used: R2-*GAL4* x UAS-*5-HT2A-RNAi* (*n* = 194 for unexposed and *n* = 193 for dead-exposed), R2-*GAL4* x *w*^*1118*^ (control; *n* = 191 for unexposed and *n* = 189 for dead-exposed), R4d-*GAL4* x UAS-*5-HT2A-RNAi* (*n* = 144 for unexposed and *n* = 134 for dead-exposed), R4d-*GAL4* x *w*^*1118*^ (control; *n* = 148 for unexposed and *n* = 143 for dead-exposed), R2/R4-*GAL4* x UAS-*5-HT2A-RNAi* (*n* = 182 for unexposed and *n* = 180 for dead-exposed), and R2/R4-*GAL4* x *w*^*1118*^ (control; *n* = 178 for unexposed and *n* = 176 for dead-exposed). (D) *foxoΔ94* mutant flies showed little lifespan differences when exposed to dead compared to unexposed flies (*n* = 184 and *n* = 180 for unexposed and dead-exposed, respectively), unlike control flies (*w*^*1118*^; *n* = 196 for unexposed and *n* = 184 for dead-exposed, respectively). (E) Knock-down of *foxo* mRNA in neurons (GS-*elav-GAL4* x UAS-*foxo-RNAi* given RU-486*; n* = 89 for unexposed and *n* = 95 for dead-exposed exposed) completely inhibited lifespan changes due to the presence of dead unlike controls (GS-*elav-GAL4* x UAS-*foxo-RNAi* without RU-486; *n* = 69 for unexposed and *n* = 95 for dead-exposed). (F) The amount of *Thor* mRNA, a Foxo target gene, was decreased in the heads of flies exposed to dead compared to unexposed flies (*n* = 16 each treatment). Plotted is the fold change in *Thor* mRNA amount isolated from fly heads when comparing the unexposed values to the dead-exposed values. (G) Foxo was required in R2/R4 neurons to see lifespan effects caused by dead exposure. The genotypes of the flies used were: R2/R4-*GAL4* x UAS-*foxo-RNAi* (*n* = 136 for unexposed and *n* = 130 for dead-exposed) and R2/R4-*GAL4* x *w*^*1118*^ (control; *n* = 136 for unexposed and *n* = 125 for dead-exposed). Data for this figure can be found in the accompanying Supporting information (S3 Datasheet). EB, ellipsoid body.

Considering that global loss of the 5-HT2A receptor abolished the effect of death exposure on lifespan and that the increased activity in EB neurons following exposure was first identified using a 5-HT2A-*GAL4* promoter, we directly tested whether *5-HT2A* expression in R2/R4 neurons is required for the effects of dead conspecifics on lifespan. We targeted RNAi-mediated suppression of *5-HT2A* expression to R2/R4 neurons and found that knockdown of *5-HT2A* in all R2/R4 neurons, as well as in R2 and R4d individually, either partially or fully reduced lifespan differences due to dead exposure (Fig 3A–3C).

### The transcription factor *foxo* is required for changes in lifespan due to death perception

To identify mechanisms that would illuminate how the EB modulates aging following death perception, we examined the requirement of signaling pathways that are known to be involved in metabolism, stress, and aging; including *Dh44* (homologue of mammalian corticotropin-releasing hormone), *NPF* (homologue of mammalian NPY), and the transcription factor *foxo*, as well as molecules involved in general aspects of fly memory (e.g., *dumb*, *rutabaga*, and *dunce*). Most of the genes or neural manipulations tested were not required (Table 2). However, we observed that flies homozygous for a null allele of *foxo* [14] did not exhibit altered lifespan when co-housed with dead (Fig 3D). A similar observation was made following RNAi-mediated knockdown of *foxo* expression in adult neurons using GS-*elav*-*GAL4* (Fig 3E). Additionally, we observed a reduction in the expression of the Foxo target gene, *Thor* (Fig 3F), in fly head tissues of individuals exposed to dead conspecifics, suggesting that Foxo activity was altered in these animals.

To determine whether Foxo is required specifically in R2/R4 neurons for death perception to affect lifespan, we targeted expression of *foxo-RNAi* to these cells (R2/R4-*GAL4* > UAS-*foxo-RNAi*). This manipulation eliminated the effect of death exposure on lifespan (Figs 3G and S3A). RNAi-mediated knockdown of *foxo* expression did not alter the appearance or number of R2/R4 neurons (S3B and S3C Fig), suggesting that our results were not caused by pleiotropic effects of RNAi on cell survival and indicating a specific function of *foxo* in mediating the effects of death perception on lifespan.

### Altered *dilp* expression in the median neurosecretory cells is observed after R4 neuronal activation

**Fig 4:**
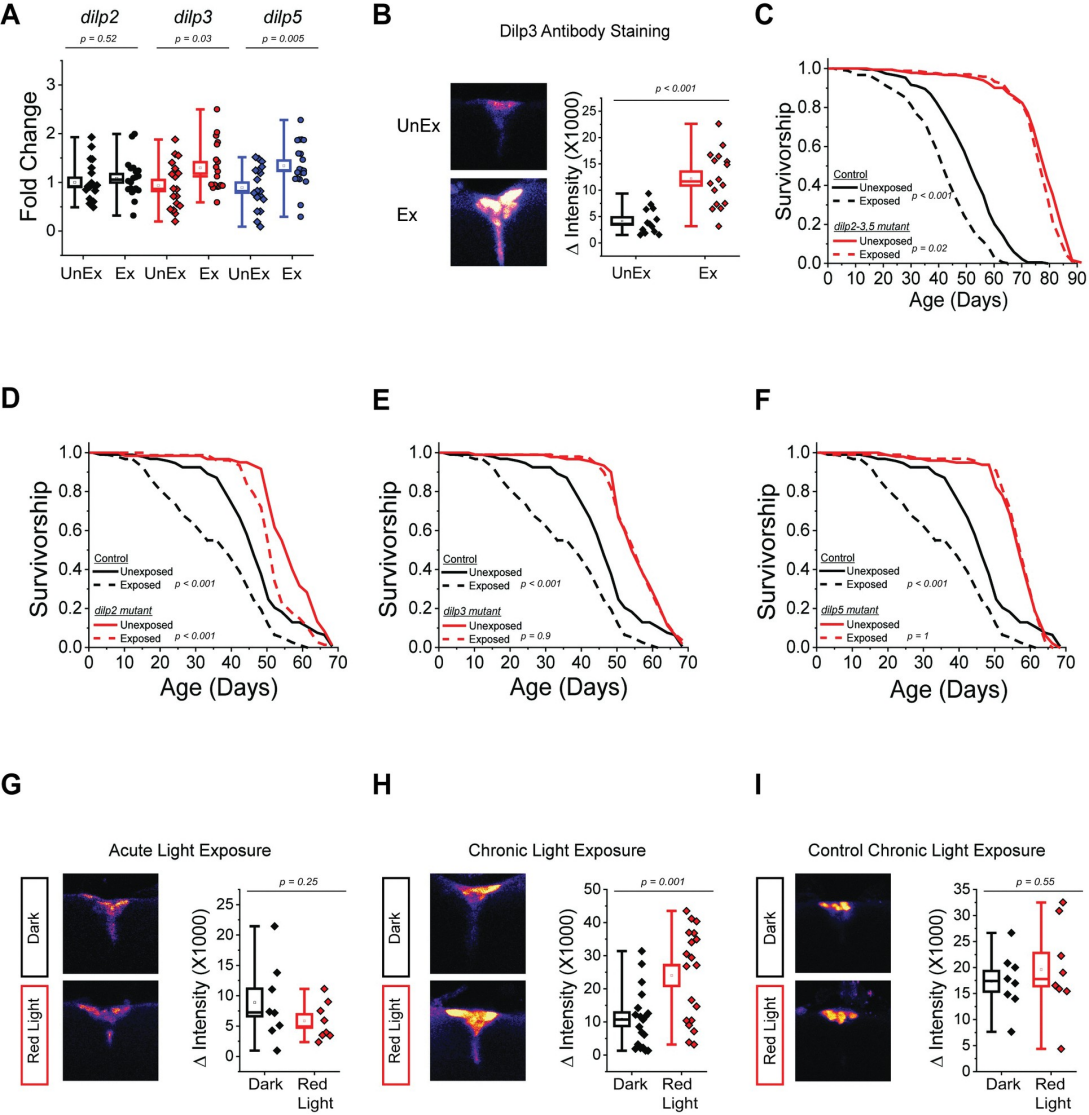
Dilps 3 and 5, but not dilp2, are required for the lifespan effects of death perception and Dilp3 protein in MNC was increased with chronic R4d activation. (A) *dilp2* mRNA was unchanged, while *dilp3* and *dilp5* mRNA abundances in Canton-S fly heads were increased upon exposure to dead conspecifics (*n* = 20 for each treatment). Plotted are the fold changes in *dilp* mRNA values when comparing the unexposed to the dead-exposed values. (B) Dilp3 protein abundance, as measured using an anti-Dilp3 antibody, was increased in Canton-S MNC upon exposure to dead conspecifics (*n* = 14 and 16 for unexposed and dead-exposed, respectively). (C) *dilp2-3*,*5* triple mutant flies (*n* = 184 for unexposed and *n* = 175 for dead-exposed) did not show altered lifespan due to the presence of dead conspecifics compared to controls (*w*^*-*^ Dahomey; *n* = 193 for unexposed and *n* = 183 for dead-exposed). (D) *dilp2* mutants showed altered lifespan when co-housed with dead conspecifics (*n* = 60 for unexposed and *n* = 79 for dead-exposed), but not *dilp3* mutants (*n* = 99 for unexposed and *n* = 90 for dead-exposed; panel E) or *dilp5* mutants (*n* = 96 for unexposed and *n* = 98 for dead-exposed; panel F) compared to controls (*w*^*1118*^; *n* = 93 for unexposed and *n* = 91 for dead-exposed). (G) Acute red light activation of R4d neurons (using R4d-*GAL4* x UAS-*Chrimson*) for 2 days had no effect on Dilp3 abundance in MNC (*n* = 8 for each treatment). (H) Chronic red light activation of R4d neurons (using R4d-*GAL4* x UAS-*Chrimson*) for 2 weeks significantly increased Dilp3 abundance in MNC (*n* = 19 for each treatment). (I) Chronic red light exposure of control flies (using R4d-*GAL4* x *w*^*1118*^) for 2 weeks had no effect on Dilp3 abundance in MNC (*n* = 8 for each treatment). Data for this figure can be found in the accompanying Supporting information (S4 Datasheet). MNC, median neurosecretory cell.

Considering that *foxo* is known to modulate aging through its role in the insulin-signaling pathway, we investigated whether the *Drosophlia* insulin-like peptides (*dilps*) were also involved in this process. Of the 8 *dilps* found in flies, we focused our studies on *dilp2*, *dilp3*, and *dilp5* because they are synthesized and released by median neurosecretory cells (MNCs) in the brain, which have previously been implicated in modulating fly lifespan [15]. We found that *dilp3* and *dilp5* mRNA, but not *dilp2* mRNA, were increased in the brains of flies that had been exposed to dead conspecifics for 2 weeks, compared to same-age, unexposed control animals (Fig 4A). Dilp3 protein abundance was also increased (Fig 4B). We were unable to examine Dilp5 protein abundance due to the lack of a Dilp5 antibody. These changes are likely important for modulating lifespan because we found that flies lacking all 3 *dilps*, or *dilp3* and *dilp5* individually, did not exhibit changes in lifespan when aged in the presence of dead conspecifics; *dilp2* was dispensable for the lifespan effect (Fig 4C–4F).

It may be that Dilp3 and Dilp5 act on R4 neurons in response to dead exposure, thereby altering *foxo* activity in these neurons that subsequently influences lifespan. R4d neurons putatively express the only insulin receptor identified in flies (InR; [16]). We therefore examined whether the expression of a dominant negative form of InR specifically in R4d neurons (R4d-*GAL4* x *InR*^*K1409A*^) inhibited lifespan changes caused by death perception. We found that this manipulation had no effect (S4 Fig), suggesting that Dilps do not act directly on R4 neurons to modulate lifespan changes due to death perception.

Alternatively, we considered the possibility that Dilp3 and Dilp5 abundances may be altered by changes in ring neuron activity to enact changes in lifespan. To evaluate this notion, we took a closer look at when molecular and phenotypic changes occur. Our data (shown in Fig 4A) demonstrated that *dilp*-expression was altered when the flies were exposed to dead for 2 weeks. We also knew that R2/R4 neurons appeared to be activated on a much shorter timescale, within 2 days (e.g., Fig 2A and 2B). We therefore asked whether *dilp3* and *dilp5* mRNA or Dilp3 protein abundance were altered after only 2 days of exposure. We observed that *dilp3* and *dilp5* mRNA abundances, as well as Dilp3 protein abundance, were unchanged following 2 days of dead exposure compared to the unexposed group (S5 Fig). These data suggest that alterations in *dilp3* and *dilp5* expression occur after modulation of R4 activity and that they may result from changes in the activity of these cells.

To test whether R4d neuron activation itself is sufficient to alter Dilp protein abundances in MNC, we used optogenetic techniques to activate R4d neurons for different lengths of time in the absence of dead conspecifics and quantified Dilp3 antibody staining. Flies expressing UAS-*Chrimson* (R4d-*GAL4* x UAS-*Chrimson*) were stimulated with red light for either 2 days or 2 weeks, after which time Dilp3 protein abundance in MNCs was quantified. We observed increased anti-Dilp3 staining when R4d neurons were activated for 2 weeks but not for 2 days (Fig 4G and 4H). Changes in Dilp3 abundance were not due to red light exposure per se because there were no changes observed in control flies (R4d-*GAL4* x *w*^*1118*^) that were exposed to a similar intensity and duration (Fig 4I). Together, these data indicate death perception rapidly activates R4d neurons, which, after a period of chronic activity, stimulate Dilp3 expression in MNC to modulate aging.

## Discussion

Here, we have identified a discrete, 5-HT2A-expressing neural population in the EB of the fly brain that plays an important role in transducing sensory information about the presence of dead individuals in the environment to influence lifespan. The R2, R4m, and R4d subpopulations were activated upon exposure to dead conspecifics, and their function was also required to modulate lifespan. Components of the insulin-signaling pathway, specifically the transcription factor Foxo in R2/R4 neurons and the insulin-like peptides Dilp3 and Dilp5, were also required to mediate the effects of death perception on lifespan. Unexpectedly, several lines of evidence support a model in which Dilps act downstream of R2/R4 neurons and therefore downstream of Foxo: (i) knockdown of the insulin receptor in R2/R4 did not impact the effects of death perception on lifespan; (ii) R4 neurons show increased calcium influx soon after dead exposure (within 48 h), while Dilp3 and Dilp5 RNA/protein abundances change over a much longer time frame (2 weeks or more; Fig 4 compared to S5 Fig); and (iii) prolonged activation of R4d neurons is sufficient to increase Dilp3 abundance in the MNC (Fig 4H; S7 Fig summarizes our findings).

Our work stimulates new questions about how distinct neural circuits impinge on well-known mechanisms of aging. How does the transcription factor Foxo act independently of insulins in *5-HT2A*^*+*^ R2/R4 neurons to transduce the effects of death perception? One possibility is that 5-HT2A signaling affects Foxo by activating downstream kinases that can phosphorylate Foxo, reducing Foxo accumulation in the nucleus and thereby altering Foxo activity [17]. 5-HT2A has previously been shown to activate phospholipase C, which can initiate the phosphoinositol second messenger cascade by producing inositol triphosphate (IP3) and diacylglycerol (DAG), stimulating the release of protein kinase C and opening L-type Ca^2+^ channels that lead to changes in neural plasticity and, hence, neural signaling [18]. Serotonin itself is known to impact Foxo activity; exogenous serotonin application down-regulated Foxo accumulation in the nucleus of wild-type *C*. *elegans* exposed to stress [19] and FoxO1 is required for serotonin to affect bone mass in mice [20]. Following death perception, it is likely that the increased intracellular Ca^2+^ levels detected in *5-HT2A*-expressing EB ring neurons resulted in greater synaptic strength as reflected by the increased intensity of Brp antibody staining. We therefore put forth a model where the sight of dead conspecifics activates 5-HT2A serotonergic receptors in EB ring neurons thereby activating this neural population through changes in both Foxo activity and greater excitability.

It is unknown how R2/R4 neurons themselves influence insulin-like peptide production and/or release. The pattern of activity generated by R2/R4 ring neurons likely propagates to ellipsoid EPG neurons [21], as these neurons synapse with R4d [16]. From here, EPG neurons synapse with a dispersed network of other neurons found in the fan-shaped body and the protocerebral bridge that could impact *dilp*-expressing MNC. Alternatively, activation of R2/R4 neurons themselves may release, perhaps in a Foxo-dependent manner, chemical messengers that travel beyond the central complex to Dilp-producing neurons. Candidate molecules whose receptors are known to be expressed on Dilp-producing MNCs and have been shown to alter their expression include short neuropeptide F [22], serotonin [23], and GABA [24]. It is also plausible that activation of R2/R4 neurons directly impacts fly metabolism, which in turn elicit secondary changes in Dilp release.

We were surprised by the observation that activation of R2 neurons alone was sufficient to significantly extend fly lifespan. Given the similar requirements of R2 and R4 neurons in the effects of death perception, one might expect that, like activation of R4, activation of R2 would also decrease lifespan. This unexpected finding indicates complexity in signaling among EB neurons and suggests that this neuropil may act as a rheostat to increase or decrease the rate of aging in response to sensory input. Understanding the similarities and differences in their downstream functions may provide insight into how this may occur. Furthermore, we are just beginning to understand the different phenotypes controlled by one or both of these neuronal groups. R2 neurons promote sleep drive [25] and increase egg-laying preference [26], whereas R4 neurons are known to promote nutritive sugar feeding [27]. Both are essential for visual orientation memory for salient objects and simple pattern discriminations [28]. Neither group has been studied for its influence on longer-term phenotypes, such as lifespan.

While the EB is generally agreed to be a multifaceted sensory integration and motor coordination center, it is one of the neuronal centers that possibly drive motivation and may even be responsible for underlying “emotion-like” states in *Drosophila*, driven mechanistically by Foxo [17,29]. A low motivational state in male flies caused by losing a fight, for example, is rescued by the optogenetic activation of the serotonin receptor 5-HT1B in EB neurons, and this state is associated with R2/R4m neural activity [30]. Moreover, the EB plays a crucial role in the stress-induced arousal response of the fly [31], a key component of many emotional and affective behaviors [32]. Lastly, there is a strong correlation in neuroanatomical organization and function between the *Drosophila* central complex and the vertebrate basal ganglia [33] suggesting that it may contribute to functions similar to those attributed to the vertebrate structure, such as recognition of emotional stimuli, inferring the emotional state of others, and awareness of subjective well-being. Foxo likely has a role in these states, as it has been suggested to play a crucial role in the pathophysiology of depressive disorders [34]. The loss of FoxO3a, for example, caused mice to display antidepressant-like behaviors [35]. Given our findings, it seems plausible that the sight of dead conspecifics elucidates a “depressive-like” state that results in decreased longevity.

While clearly speculative, conceptual distinctions of neural states reflect causal influences on organism health, and they have real-world consequences that are clinically tangible. Death perception may be one of these, as it produces psychological changes in humans such as emotional dysregulation and depression, as well as important physiological changes that negatively impact overall health, including depression, headache, fatigue, and cardiovascular disease [36–38]. Could motivational therapy or pharmacologic intervention in reward systems, much like what is done for addiction, slow aging? Such ideas are testable today, in humans, using approved drugs once we have a clearer mechanistic insight into the neural circuits and signaling systems that are involved.

## Materials and methods

### Experimental model and subject details

The origin details about the laboratory stocks used in this paper can be found in S1 Table. Of note, the GS*-elav-GAL4* line used herein contains 3 copies of the GS-*elav-GAL4* insertion (1 on the second and 2 on the third chromosome) and was created using standard genetic *Drosophila* techniques in our laboratory by B. Chung. The expression patterns of the R2-*GAL4*, R4m-*GAL4*, R4d-*GAL4*, and R2/R4-*GAL4* drivers used in this study were visualized by crossing each line to UAS-*GFP* and resembled previously published expression patterns [8]. All of the mutant lines used herein that target a specific gene (e.g., *foxoΔ94*, *dilp2*, etc.) were homozygous for the mutation.

### Generation of dead flies

Dead flies were generated using starvation. Briefly, approximately 30 Canton S flies (of both sexes and 1 to 2 weeks of age) were placed into vials that contained 2% agar + 0.3% Tegosept media. The flies were placed in an 25°C incubator with 12:12 h light-dark cycles. Dead flies used for the dead-exposed treatments were collected for experimental use within 3 days of their death (termed “freshly dead”).

### Exposure to dead flies

Twenty mated female flies were collected under light CO_2_ anesthesia and exposed to 14 freshly dead flies in standard food, where they freely interact with the dead flies for the designated time period (anywhere from 2 days to their whole life, as indicated). During the exposure period, flies were maintained in a 12:12 h light-dark cycle, at 25°C and 60% relative humidity. For exposures longer than 2 days, the flies that were exposed to dead were flipped onto fresh food containing 14 freshly dead flies every 2 to 3 days for their entire exposure time. Unexposed, control flies (those without fresh dead) were also flipped onto fresh food every 2 to 3 days at the same time as the exposed flies.

### Survival analysis

For the lifespan experiments, control and experimental flies were reared under controlled larval density at 25°C and collected as adults within 24 h of emergence onto standard food where they mated freely for 2 to 3 days (see S6 Fig for a cartoon description of the experimental setup for our lifespan assays). At 3 days post-eclosion, female flies were sorted under light CO_2_ and then placed into fresh food vials. Lifespan measures were obtained using well-established protocols [2]. Flies were transferred to fresh food vials every Monday, Wednesday, and Friday, at which time 14 freshly dead flies (approximately 1 to 3 days postmortem) were added. Unexposed animals were transferred simultaneously, but instead of adding dead flies, we removed any flies that had died since the last census time. Treatment flies were therefore exposed continuously to at least 14 dead flies throughout their life. Control flies, on the other hand, did not see dead until they started dying themselves (often after 40 to 50 days of adult life), and these few dead were removed from the vials every Monday, Wednesday, and Friday. Flies were maintained at 25°C and 60% humidity under a 12:12 h light-dark cycle. For experiments using UAS-*Chrimson*, flies were exposed to red light (630 nm) at 40 Hz (32% duty cycle) and a power of 2,060 lux during the 12 h lights-on time. For experiments using UAS-*GtACR1*, flies were exposed to green light (520 nm) at 40 Hz (32% duty cycle) and a power of 3,540 lux during the 12 h lights-on time. For experiments using UAS-*dTRPA1*, the flies were reared and held at 18°C until the lifespans were set up, when they were placed at 29°C.

While it is widely known that genetic background can significantly impact lifespan, nearly all of our experiments derived inference from comparisons within background. To be clear, when testing for requirement we compared flies of the exact same genotype that were divided between the unexposed and dead-exposed groups. In experiments where we compared across genotypes (e.g., Fig 2C–2F), we use *GAL4* flies that were crossed to the UAS line that was backcrossed for a minimum of 10 generations.

### qPCR

All flies for qPCR analysis were flash frozen in liquid nitrogen for storage at −80°C. Fly heads were collected by vortexing the frozen flies in a 15 ml conical tube for 15 s, plunging them in liquid nitrogen, and then separating them from their bodies using a chilled metal sieve. Total mRNA was extracted from 30 fly heads in FastPrep Lysing Matrix D tubes (MP Biomedicals) using 500 ml cold TRIzol (Thermo Fisher Scientific) according to the manufacturer’s directions. The RNA-containing fraction (the top 100 μl) was then removed by pipet to a fresh, autoclaved microcentrifuge tube for RNA precipitation using 200 μl 100% ethanol. After 30 min at −80°C, the RNA pellet was collected by spinning at 13,000 rpm in a benchtop centrifuge. The pellet was washed 2× using 70% ethanol in DEPC water and then dissolved in 20 μl DEPC water for spectrophotometer analysis at 260 nm. Equal amounts of RNA from each sample were subjected to reverse transcription using SuperScript III RT enzyme and protocol (Invitrogen) according to the manufacturer’s directions. The subsequent cDNA was then subjected to qPCR analysis in a StepOne Plus 96-well Thermocycler (Applied Biosystems) using SYBR Green PCR Master Mix (Applied Biosystems) according to the manufacturer’s instructions. The primer sequences used for amplification are as follows:

dilp2

Forward primer: 5′ TCTGCAGTGAAAAGCTCAACGA 3′; Reverse primer: 5′ TCGGCACCGGGCATG 3′

dilp3

Forward primer: 5′ AGAGAACTTTGGACCCCGTGAA 3′; Reverse primer: 5′ TGAACCGAACTATCACTCAACAGTCT 3′

dilp5

Forward primer: 5′ GAGGCACCTTGGGCCTATTC 3′; Reverse primer: 5′ CATGTGGTGAGATTCGGAGCTA 3′

Rpl32

Forward primer: 5′ CGGATCGATATGCTAAGCTGT 3′; Reverse primer: 5′ GCCCTTGTTCGATCCGTA 3′

*Thor* (*4E-BP*)

Forward primer: 5′ TGCCCATGATCACCAGGAAG 3′; Reverse primer: 5′ CATGAAAGCCCGCTCGTAGA 3′.

### NFAT experiments

For NFAT exposure experiments, the flies were collected within a few hours of eclosion and kept in vials with standard food. On the second day, female flies were separated quickly from the male flies on a cold pad and then were put into vials with or without 14 fresh dead for another 2 days. On day 3, the female brains were dissected and imaged as described below using a wavelength of 488 nm to visualize GFP.

### CaMPARI experiments

Mated, 10-day-old female flies were collected by brief exposure to CO_2_ and placed into vials containing standard food with or without dead (see exposure to dead flies above for details). At the end of the 2-day exposure, the flies were transferred to a small light-exposure chamber with or without dead flies and allowed to acclimate for 1 h. The flies were then exposed to photo convertible light (405 nm) for 1.5 h at room temperature at 2 Hz (40% duty cycle) using a power of at least 75 mW/cm^2^. After the exposure, the flies were briefly exposed to CO_2_ and sorted into groups of 2, placing each group into a small Eppendorf tube at which point the treatments were randomized. The flies were subsequently visually examined to ensure both were alive. The flies were then placed on ice, their brains dissected, and then imaged as described below using wavelengths of 488 nm (for green) and 594 nm (for red).

### Brain dissection

Adult female brains from exposed or unexposed conditions were dissected in ice-cold phosphate buffer saline (PBS) using sharpen tweezers and fixed in PBS containing 4% paraformaldehyde for 60 min at room temperature. The brains were then moved using a wide-bore pipet tip to either a glass slide with Vectashield mounting medium (ZH0219) and sealed using a coverslip and nail polish for immediate 488 nm imaging or to a microcentrifuge tube to undergo the immunostaining protocol, described below.

### Immunostaining

Brains undergoing the immunostaining protocol were first allowed to sink to the bottom of the microcentrifuge tube containing 1 ml of PBS with 0.1% Triton-X (PBS-T), the supernatant was removed, and then 1 ml of fresh PBS-T solution was added (quick wash). After 3 quick washes, the brains were washed an additional 3 times using a slightly longer wash protocol (for each wash, 1 ml PBS-T was added and the microcentrifuge tube was placed on the orbital shaker for 20 min). After the last wash, the PBS-T solution was removed, and a block solution containing 5% normal goat serum (Caymen Chemical Company) was added. The samples were placed on the orbital shaker for 30 min at room temperature.

For CaMPARI immunostaining, the block solution was removed and a 1:1,000 dilution in 5% normal goat serum of the anti-CaMPARI-Red antibody was added (Absolute Antibody). The brains were allowed to mix gently on the orbital shaker at 4°C for 4 days. On day 5, the primary antibody solution was removed, and the brains were subjected to 3 quick washes with PBS-T. After these quick washes, the samples were subjected to 3 longer, 20-min washes, each with 1 ml PBS-T. Next, a 1:500 dilution of anti-rabbit Alexa Fluor 594 (Invitrogen) in 5% normal goat serum was added. Each microcentrifuge tube was covered with tin foil at this step to preserve the fluorescence of the secondary antibody. The tubes were then placed on the orbital shaker at 4°C for 1 night. The next day, the secondary antibody was removed, and 3 quick washes followed by 3 longer, 20-min washes were performed using PBS-T. After the last wash, the brains were transferred to a glass slide with Vectashield mounting medium (ZH0219) using a wide-bore pipet tip and sealed with coverslips and nail polish.

For the Bruchpilot or Dilp3 immunostaining, the nc82 antibody (Developmental Studies Hybridoma Bank) or the Dilp3 antibody (gifted by J. Veenstra) was added at a 1:50 or 1:250 dilution, respectively, in 5% normal goat serum and incubated at 4°C for 3 days. On day 4, the brains were washed using PBS-T 4 times for 15 min each at room temperature and then incubated with secondary antibody for 24 h at room temperature (Mouse Alexa Fluor 568 or Rabbit Alexa Fluor 568 at a dilution of 1:500 in 5% normal goat serum, respectively). The next day, the secondary antibody was removed, and 3 quick washes followed by 3 longer, 20-min washes were performed using PBS-T. After the last wash, the brains were transferred to a glass slide with a wide-bore pipet tip in Vectashield mounting medium (ZH0219) and sealed with coverslips and nail polish.

### Imaging and analysis

Imaging was carried out using an Olympus FLUOVIEW FV3000 confocal microscope. The brains were brought into focus with a 10× (0.40 NA) objective lens before switching to 20× (0.75 NA). Images were acquired at 1,024 × 1,024 pixels with a step size of 3.0 micron; extra care was taken in order to not saturate an image. The laser power and the parameters for image acquisition were kept similar between control and treatment groups.

For data analysis, the imaging files were analyzed using the publicly available imaging software, Fiji. First, the split channel was selected to open the individual channels of each image (red and green). Selective slices were combined and collapsed into a single image using SUM slices. Brightness and contrast were adjusted manually and in a similar manner across all treatments when required for better visualization of the image. Background was calculated from the brain region adjacent to the ROI and subtracted from each channel, i.e., (Green channel-Background) and (Red channel-Background) and is plotted as the change in fluorescence intensity (Δ Intensity). For CaMPARI imaging, the resultant ratios of (RFP-Background)/(GFP-Background) were calculated and plotted.

### Statistical analysis

For lifespan, we employed survival analysis. Unless otherwise indicated, group- and pairwise-comparisons among survivorship curves were performed using the DLife computer software [39] and the statistical software R. *P*-values were obtained using log-rank analysis. For all box plots, the box represents standard error of the mean (SEM, centered on the mean), the whiskers represent the min and max values, the line represents the median, and the square dot represents the mean. For qPCR, we used the Delta-Delta CT method to present our mRNA quantifications. Briefly, ΔCT values were calculated by taking the measured CT value of the gene of interest and subtracting its corresponding RPL32 CT value that was used as a control for the amount of cDNA loaded. Each ΔCT was then transformed by 2^^-(ΔCT)^. The 2^^-(ΔΔCT)^, or fold change, was calculated by dividing each individual 2^^-(ΔCT)^ value by the average 2^^-(ΔCT)^ value of the unexposed group. The data were then analyzed using a standard *t* test with Welch’s correction. For Δ Intensity plots, *P*-values were obtained by standard two-sided *t* test after verifying normality and equality of variances. If variances were unequal, then a Welch correction was performed.

## Supporting information

S1 Fig Inhibition of TuBu neurons inhibited changes in lifespan due to the presence of dead. (A) The GFP Expression pattern of TuBu-*GAL4* used is shown (TuBu-*GAL4* x 20XUAS-IVS-*mCD8*::*GFP*). (B) The genotypes of the flies used were: TuBu-*GAL4* x UAS-*KIR*^*2*.*1*^ (*n* = 197 for unexposed and *n* = 177 for dead-exposed) and TuBu-*GAL4* x *w*^*1118*^ (control: *n* = 189 for unexposed and *n* = 180 for dead-exposed). (C) The panel on the left are representative brain images from selective slices of R2/R4-*GAL4* x NFAT unexposed and dead-exposed flies kept in the dark. The panel on the right is the quantification of the GFP-intensity seen in the ellipsoid cell bodies and axons from the regions indicated the picture of unexposed and dead-exposed brains. The pixel sum of all the stacks from the indicated region with background subtracted is shown. Each dot represents an individual brain (*n* = 8 brains for each treatment). Data for this figure can be found in the accompanying Supporting information (S1 Data).
(EPS)

S2 Fig Maintaining EB-*GAL4* x UAS-*TRPA1* flies at the permissive temperature (18°C) had no effect on their lifespan. The genotypes and numbers used were EB-*GAL4* x UAS-*TRPA1* (*n* = 136) and EB-*GAL4* x *w*^*1118*^ (*n* = 128). Data for this figure can be found in the accompanying Supporting information (S2 Data).
(EPS)

S3 Fig Knock-down of *foxo* inhibited changes in lifespan due to death perception and had no obvious effect on cell morphology or number. (A) Knock-down of *foxo* in R4d neurons (R4d-*GAL4* x UAS-*foxo-RNAi*, *n* = 130 for unexposed and *n* = 123 for dead-exposed) inhibited lifespan changes caused by the presence of dead compared to the control (R4d-*GAL4* x *w*^*1118*^, *n* = 133 for unexposed and *n* = 130 for dead-exposed). Using R2/R4-*GAL4* x UAS-*GFP*; UAS-*foxo-RNAi*, there was no visible difference in ring cell morphology (B) or cell number (C) compared to the control cross (R2/R4-*GAL4* x UAS-*GFP*). For each treatment, *n* = 8. Data for this figure can be found in the accompanying Supporting information (S3 Data).
(EPS)

S4 Fig A functional insulin receptor was not required on R4d neurons to mediate the lifespan effects of death perception. The genotypes of the flies used for this experiment are R4d-*GAL4* x *w*^*1118*^ (control; *n* = 159 for unexposed and *n* = 144 for dead-exposed) and R4d-*GAL4* x UAS-*InR*^*K1409A*^ (*n* = 156 for unexposed and *n* = 137 for dead-exposed). Data for this figure can be found in the accompanying Supporting information (S4 Data).
(EPS)

S5 Fig *dilp2*, *dilp3*, and *dilp5* mRNA, as well as Dilp3 protein abundances, were unchanged when flies were exposed to dead for 2–3 days. (A) *dilp2*, *dilp3*, and *dilp5* mRNA abundances in heads were unchanged when Canton-S flies were exposed to dead conspecifics compared to their unexposed siblings (*n* = 5 for each treatment). (B) Representative images of brains from unexposed and dead-exposed flies (R4d-*GAL4* x UAS-*Chrimson*) stained with an anti-Dilp3 antibody. On the right is a quantification of the anti-Dilp3 antibody staining in unexposed compared to dead-exposed flies (*n* = 8 for each treatment). Data for this figure can be found in the accompanying Supporting information (S5 Data).
(EPS)

S6 Fig Cartoon depicting fly husbandry and data collection for lifespans. Briefly, flies were placed into a small, 6 cm diameter cage that has a petri plate at the bottom filled with grape juice agar (Genesse Scientific). The grape juice agar-containing plate was changed every 16–20 h. *Drosophila* eggs were gently collected from the grape juice agar plate using PBS and poured into a 15 ml conical tube where they settled to the bottom of the tube. After 5 min, the PBS was poured off, and the eggs were washed with an additional PBS rinse. After a second 5-min period, the PBS was poured off and the eggs were aliquoted (32 μl) into bottles containing standard *Drosophila* food. The bottles were placed into a 25°C incubator with 12 h:12 h light:dark cycles and the flies were allowed to develop over 10 days. When the flies emerged, they were collected into fresh bottles containing standard *Drosophila* food and allowed to mate for 2–3 days. A portion of the flies were then placed into vials containing 2% agar-only; these flies died over the next few days and were used as our fresh dead fly population (dead bank). The rest of the flies were briefly gassed with CO_2_ and females were placed into vials containing standard *Drosophila* food, 20 per vial. Vials that had food alone were the control condition, while vials that had both food and 14 fresh dead flies were the experimental condition. The food/dead flies were changed every Monday, Wednesday, and Friday. While changing their food, we removed the number of live flies that died from each vial and put number this into our Dlife software program. This figure was created with BioRender.com.
(EPS)

S7 Fig Cartoon summary of our data detailing how death perception impacts fly lifespan. This figure was created with BioRender.com.
(EPS)

S1 Table Fly lines used in the manuscript. (EPS)

S1 Datasheet (A) The values shown are the change in the fluorescence intensity of the regions indicated in Fig 1A minus the background fluorescence for each brain analyzed. The genotype of the flies used here was *5-HT2A-GAL4* x NFAT. (B) The values shown are the change in the fluorescence intensity of the regions indicated in Fig 1B minus the background fluorescence for each brain analyzed. The genotype of the flies used here was EB-*GAL4* x NFAT. (C–F) The first column is the age of the animals in days, while each subsequent column contains the survivorship values calculated for the fly population indicated at the top. The genotypes of the flies used were: (C) EB-*GAL4* x *w*^*1118*^ (control) or EB-*GAL4* x UAS-*Kir*^*2*.*1*^ (EB neurons inhibited), (D) R4d-*GAL4* x *w*^*1118*^ (control) or R4d-*GAL4* x UAS-*Kir*^*2*.*1*^ (R4d neurons inhibited), (E) R2-*GAL4* x *w*^*1118*^ (control) or R2-*GAL4* x UAS-*GtACR1* (R2 neurons inhibited), and (F) R4m-*GAL4* x *w*^*1118*^ (control) or R4m-*GAL4* x UAS-*GtACR1* (R4m neurons inhibited).
(XLSX)

S2 Datasheet (A) The values shown are the change in the fluorescence intensity of the regions indicated in Fig 2A for each brain analyzed. This value is calculated as the intensity of RFP fluorescence (neurons that show increased Ca^2+^ influx)/the intensity of GFP fluorescence (all neurons that express the CaMPARI construct). (B) The values shown are the change in the anti-Bruchpilot antibody staining intensity of the regions shown in Fig 2B minus the background fluorescence for each brain analyzed. (C–F) The first column is the age of the animals in days, while each subsequent column contains the survivorship values calculated for the fly population indicated at the top. The genotypes of the flies used were: (C) EB-*GAL4* x *w*^*1118*^ (control) or EB-*GAL4* x UAS- *TRPA1* (EB neurons activated), (D) R2/R4d-*GAL4* x *w*^*1118*^ (control) or R2/R4d-*GAL4* x UAS-*TRPA1* (R2/R4d neurons activated), (E) R4d-*GAL4* x *w*^*1118*^ (control) or R4d-*GAL4* x UAS-*TRPA1* (R4d neurons activated), and (F) R2-*GAL4* x *w*^*1118*^ (control) or R2-*GAL4* x UAS-*TRPA1* (R2 neurons activated).
(XLSX)

S3 Datasheet (A–E) The first column is the age of the animals in days, while each subsequent column contains the survivorship values calculated for the fly population indicated at the top. The genotypes of the flies used were: (A) R2-*GAL4* x *w*^*1118*^ (control) or R2-*GAL4* x UAS-*5-HT2A-RNAi*, (B) R4d-*GAL4* x *w*^*1118*^ (control) or R4d-*GAL4* x UAS-*5-HT2A-RNAi*, (C) R2/R4-*GAL4* x *w*^*1118*^ (control) or R2/R4-*GAL4* x UAS-*5-HT2A-RNAi*, (D) *w*^*1118*^ (control) or *foxoΔ94*, (E) GS-*elav-GAL4* x UAS-*foxo-RNAi* without RU-486 (control) or GS-*elav-GAL4* x UAS-*foxo-RNAi* given RU-486. (F) Shown are the fold changes in *Thor* mRNA amounts collected from the heads of flies that were either unexposed or dead-exposed. (G) The first column is the age of the animals in days, while each subsequent column contains the survivorship values calculated for the fly population indicated at the top. The genotypes of the flies used were R2/R4-*GAL4* x *w*^*1118*^ (control) or R2/R4-*GAL4* x UAS-*foxo-RNAi*.
(XLSX)

S4 Datasheet (A) Shown are the fold changes in *dilp2*, *dilp3*, or *dilp5* mRNA amounts collected from the heads of flies that were unexposed or dead-exposed for 14 days. (B) The values shown are the change in the anti-Dilp3 antibody staining intensity of the MNC in the brains of flies that were unexposed or dead-exposed for 14 days minus the background fluorescence for each brain analyzed. (C–F) The first column is the age of the animals in days, while each subsequent column contains the survivorship values calculated for the fly population indicated at the top. The genotypes of the flies used were: (C) *w*^*-*^ Dahomey (control) or *dilp2-3*,*5* triple mutant flies, (D) *w*^*1118*^ (control) or *dilp2* mutant flies, (E) *w*^*1118*^ (control) or *dilp3* mutant flies, and (F) *w*^*1118*^ (control) or *dilp5* mutant flies. (G–I) The values shown are the change in the anti-Dilp3 antibody staining intensity of the MNC, minus the background fluorescence for each brain analyzed. The treatments consisted of the following: (G) R4d-*GAL4* x UAS-*Chrimson* flies kept in the dark (control) or given an acute (2 days) red light exposure, (H) R4d-*GAL4* x UAS-*Chrimson* flies kept in the dark (control) or given a chronic (14 days) red light exposure, and (I) R4d-*GAL4* x *w*^*1118*^ flies kept in the dark (control) or given a chronic (14 days) red light exposure.
(XLSX)

S1 Data (B) The first column is the age of the animals in days, while each subsequent column contains the survivorship values calculated for the fly population indicated at the top. The genotypes of the flies used were: TuBu-*GAL4* x *w*^*1118*^ (control) or TuBu-*GAL4* x UAS-*KIR*^*2*.*1*^. (C) The values shown are the change in the fluorescence intensity of the regions indicated in S1C Fig minus the background fluorescence for each brain analyzed. The genotype of the flies used here was R2/R4-*GAL4* x NFAT and all of the flies were kept in the dark.
(XLSX)

S2 Data The first column is the age of the animals in days, while each subsequent column contains the survivorship values calculated for the fly population indicated at the top. The genotypes of the flies used were EB-*GAL4* x *w*^*1118*^ (control) or EB-*GAL4* x UAS-*TRPA1*. The flies were aged at 18°C.
(XLSX)

S3 Data (A) The first column is the age of the animals in days, while each subsequent column contains the survivorship values calculated for the fly population indicated at the top. The genotypes of the flies used were R4d-*GAL4* x *w*^*1118*^ or R4d-*GAL4* x UAS-*foxo-RNAi*. (C) The numbers of GFP-positive cells counted per brain in R2/R4-*GAL4* x *UAS-GFP* (control) or R2/R4-*GAL4* x UAS-*GFP*; UAS-*foxo-RNAi* animals.
(XLSX)

S4 Data The first column is the age of the animals in days, while each subsequent column contains the survivorship values calculated for the fly population indicated at the top. The genotypes of the flies used were R4d-*GAL4* x *w*^*1118*^ (control) or R4d-*GAL4* x UAS-*InR*^*K1409A*^.
(XLSX)

S5 Data (A) Shown are the fold changes in *dilp2*, *dilp3*, or *dilp5* mRNA amounts when flies were unexposed or dead-exposed for 2–3 days. (B) The values shown are the change in the anti-Dilp3 antibody staining intensity of MNC minus the background fluorescence for each brain analyzed for flies that were unexposed or dead-exposed for 2–3 days.
(XLSX)

## Abbreviations

CNS: central nervous system
EB: ellipsoid body
GFP: green fluorescence protein
MNC: median neurosecretory cell
PBS: phosphate buffer saline
RFP: red fluorescence protein
TuBu: tubercular-bulbar
